# Chronic Right Heart Failure: Pathogenesis, Haemodynamic Foundations, and a Pragmatic Diagnostic Algorithm

**DOI:** 10.3390/jcdd13070317

**Published:** 2026-07-09

**Authors:** Frank Lloyd Dini, Alberto Palazzuoli, Erberto Carluccio, Gian Marco Rosa, Michele Ciccarelli, Valentina Mercurio, Gaetano Ruocco, Andrea Salzano, Michele Correale, Stefano Ghio, Stefania Paolillo, Savina Nodari, Gianfranco Sinagra

**Affiliations:** 1 Auxologico: Istituto di Ricovero e Cura a Carattere Scientifico (IRCCS), 20149 Milano, Italy; 2 Cardiovascular Diseases Unit, Cardio Thoracic and Vascular Department, AOUS, University of Siena, 53100 Siena, Italy; palazzuoli2@unisi.it; 3 Cardiology and Cardiovascular Pathophysiology, University Hospital S. Maria della Misericordia of Perugia, Piazzale Menghini 1, 06129 Perugia, Italy; erberto.carluccio@unipg.it; 4 Department of Internal Medicine, University of Genova, 16126 Genova, Italy; gian.marco.rosa@unige.it; 5 Departmentof Medicine, Surgery and Dentistry, University of Salerno, 84081 Salerno, Italy; mciccarelli@unisa.it; 6 Department of Translational Medical Sciences, Federico II University, 80138 Naples, Italyandrea.salzano@unina.it (A.S.); 7 Cardiology Unit, “I. Veris Delli Ponti” Hospital, ASL Lecce, 73020 Scorrano, Italy; gaetanomaria.ruocco@asl.lecce.it; 8 Department of Cardiology, University Hospital Foggia, 71122 Foggia, Italy; michele.correale@unifg.it; 9 Division of Cardiology, Fondazione IRCCS Policlinico S. Matteo, 27100 Pavia, Italy; s.ghio@smatteo.pv.it; 10 Department of Advanced Biomedical Sciences, Federico II University of Naples, 80131 Napoli, Italy; stefania.paolillo@unina.it; 11 Cardiology Section, Department of Medical and Surgical Specialities, Radiological Sciences and Public Health, University of Brescia, Cardiothoracic Department, Civil Hospitals, 25123 Brescia, Italy; savina.nodari@unibs.it; 12 Centre for Diagnosis and Treatment of Cardiomyopathies, Cardiovascular Department, Azienda Sanitaria Universitaria Giuliano-Isontina (ASUGI), University of Trieste, 34149 Trieste, Italy

**Keywords:** chronic right heart failure, central venous pressure, right ventricular-pulmonary artery coupling, systemic congestion, echocardiography, diagnostic algorithm

## Abstract

Chronic right heart failure (RHF) is a complex, progressive syndrome that remains underrecognized and inadequately defined in current clinical guidelines, where it is often relegated to a secondary complication of left-sided heart disease. Because the thin-walled right ventricle (RV) is well adapted to maintain pressures within the highly distensible venous system well below plasma oncotic pressure but poorly equipped to sustain pressure overload, when myocardial failure supervenes, conventional RV systolic indices frequently fail to capture the very essence of the syndrome. This review clarifies the distinct pathophysiological and haemodynamic foundations of chronic RHF, framing it fundamentally as the heart’s inability to decongest the systemic venous circulation. We highlight how backward failure, rather than isolated RV systolic dysfunction, drives systemic and multi-organ congestion. To bridge existing diagnostic gaps, we propose a pragmatic, diagnostic algorithm. Under this framework, a Definite Diagnosis of chronic RHF requires evidence of elevated right atrial/central venous pressures—defined as clinically raised jugular venous pressure plus an echocardiographic dilated inferior vena cava (>21 mm with <50% collapse)—alongside at least one of four minor criteria: (1) systemic or visceral congestion (e.g., persistent oedema, congestive hepatomegaly); (2) echocardiographic RV systolic dysfunction (TAPSE < 17 mm, FAC < 35%, S′ < 9.5 m/s; RV free-wall strain > −20%); (3) non-invasive signs of pulmonary hypertension (TRV > 2.8 m/s); or (4) impaired RV-pulmonary arterial coupling (TAPSE/PASP ratio < 0.35). By centering diagnosis on systemic venous hypertension as a result of right heart backward failure rather than isolated RV metrics, this framework offers a coherent, readily applicable tool for diagnosing chronic RHF in routine clinical practice.

## 1. Introduction

Heart failure (HF) is a highly heterogeneous clinical syndrome characterised by a broad spectrum of symptoms and physical signs [1]. Over time, several classifications have been proposed to better characterise individual patients and guide optimal management. HF has traditionally been defined as a condition in which the heart is unable to pump blood at a rate sufficient to meet the metabolic demands of the tissues, or can do so only at the expense of elevated filling pressures [2]. More recently, HF has been described as a syndrome identified by symptoms and signs caused by structural and/or functional cardiac abnormalities, supported by elevated natriuretic peptide (NP) levels and/or objective evidence of pulmonary or systemic congestion [3].

Within this broad spectrum, right HF (RHF) represents a distinct and often under-recognised entity [4,5,6]. Importantly, RHF does not arise from a single disease process but rather from three major categories, each characterised by specific pathogenetic mechanisms, clinical manifestations, and diagnostic implications:

1. Pulmonary vascular disease-related RHF, driven primarily by chronic pressure overload due to pulmonary vascular obstruction or remodelling (including acute and chronic cor pulmonale and pulmonary arterial hypertension: PAH).
2. Left heart disease-related RHF, the most common form, resulting from the backward transmission of elevated left-sided filling pressures.
3. Primary right ventricular (RV) disorders, in which intrinsic myocardial injury, cardiomyopathy, or inflow/outflow obstruction directly impair RV performance.

Although these processes differ substantially in their upstream mechanisms, they ultimately converge on a shared downstream haemodynamic phenotype characterised by progressive right atrial (RA)/central venous (CV) pressure elevation, RV–pulmonary arterial uncoupling, systemic venous congestion and—when RV contractile reserve becomes exhausted—reduction in forward flow. Despite this common final pathway, RHF is still frequently described using definitions that mirror the historical framework of left-sided HF (LHF), often overlooking the unique anatomical, physiological, and RV-arterial coupling characteristics of the RV [4]. While a universally accepted definition remains elusive, diagnostic criteria continue to be heterogenous. This gap has been partly addressed by the 2024 Heart Failure Association (HFA) and the European Association of Percutaneous Cardiovascular Interventions (EAPCI) of the ESC clinical consensus statement [7], which advocates shifting the clinical focus from isolated RV systolic indices to systemic backward venous hypertension and target-organ venous congestion. However, a standardized, criteria-driven system capable of defining hierarchical diagnostic boundaries across the spectrum of clinical and instrumental features related to RHF remains an unmet clinical need.

This review delineates the shared haemodynamic mechanisms and diagnostic principles of chronic RHF, offering a unifying mechanistic framework rather than an aetiologic catalogue. While acknowledging the many disease-specific causes of RHF, we focus on the common pathway of elevated right-sided filling pressures and integrate this haemodynamic hallmark with clinical and echocardiographic markers of systemic venous congestion and RV dysfunction and overload to propose a pragmatic, clinically applicable diagnostic algorithm.

## 2. Pathogenesis and Pathophysiology

Although RHF shares several pathogenetic mechanisms with LHF, its clinical expression is shaped by the unique structural and functional properties of the RV and its tight coupling with the pulmonary circulation [8]. The thin-walled, highly compliant RV is well adapted to handle volume but poorly equipped to sustain pressure overload. Under physiological conditions, this anatomical and physiological peculiarity enables the RV to maintain pressures within the highly distensible venous system at levels well below plasma oncotic pressure, thereby ensuring optimal venous return and adequate tissue perfusion [9,10,11].

Increased afterload is a major determinant of RV dysfunction (RVD) and failure [12,13]. Right-sided pressure overload and RVD are the fundamental pathogenetic mechanisms responsible for RHF; as they are intrinsically linked, they often characterise the syndrome simultaneously [14]. An increase in pulmonary artery pressure (PAP) of 20 mmHg has been shown to reduce RV stroke volume by approximately 30% [15]. Pulmonary hypertension (PH) therefore leads to RVD with or without RV dilation [16]. RHF may also arise in patients with PAP within normal limits, either because of primary RV pathology or because the RV has exhausted its ability to compensate for increased loading conditions. Although RVD and RHF frequently coexist, they are not synonymous. Some patients display impaired RV systolic function without clinical signs of RHF, whereas others develop RHF despite preserved—or apparently preserved—RV systolic performance. When contractile impairment is present, RV output may be maintained through compensatory chamber dilation. Conversely, RHF may arise in patients with moderate-to-severe tricuspid regurgitation (TR) in whom RV systolic indices may still appear within the normal range. Therefore, the terms RVD and RHF should not be used interchangeably [17].

**Figure 1:**
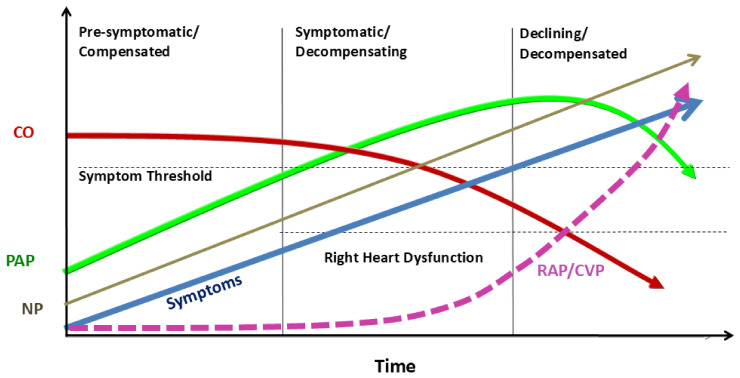
Schematic representation of the progressive transition from a pre-symptomatic, compensated state to overt, decompensated chronic right heart failure (RHF) over time. (**Top**) Changes in pulmonary artery pressure (PAP, green curve) and cardiac output (CO, orange curve). Initially, PAP rises while CO is preserved via homeostatic mechanisms. As right ventricular (RV) dysfunction advances past the symptom threshold, a precipitous decline in CO occurs alongside a stabilization or paradoxical drop in PAP, signifying forward failure. (**Bottom**) Corresponding trends in right atrial pressure/central venous pressure (RAP/CVP, red curve) and natriuretic peptides (NP, grey curve). Overt clinical symptoms emerge when RAP/CVP elevate significantly, reflecting a profound loss of the RV’s capacity to preserve low systemic venous pressures.

As detailed in Table 1 and Table 2, chronic RHF can be caused or triggered by a wide array of distinct clinical inputs. The temporal evolution of PAP, cardiac output (CO), and RA/CV pressures from pre-symptomatic compensated RV overload and/or RVD to overt RHF is illustrated in Figure 1. If the right heart compensates for pressure overload but fails to decongest the systemic venous system, excessively high RA/CV pressures and systemic congestion ensue. While the RV can tolerate overload for extended periods, it—much like the left ventricle (LV)—will eventually fail.

## 3. Acute Versus Chronic Right Heart Failure

RHF may develop acutely or chronically. Two forms of acute RHF are recognized (Table 3): newly arisen (“de novo”) RHF and acutely decompensated chronic RHF. Acute RHF most often occurs when the overloaded RV fails to maintain adequate forward flow into the pulmonary circulation [18,19].

Acute RHF is typically characterised by rapid forward failure and hypoperfusion, as seen in massive pulmonary embolism. In the acute setting, the non-preconditioned RV cannot tolerate a sudden increase in afterload. This mismatch often precipitates abrupt RV failure, potentially leading to rapid chamber dilation, an interventricular septal shift, and ultimately a catastrophic drop in CO (obstructive shock) [20,21,22,23]. In acutely decompensated chronic RHF, hypoperfusion develops in the context of chronic backward failure of the RV.

When RV overload develops gradually—allowing time for structural and functional adaptation—the RV may initially compensate despite chronically increased afterload. Increased outflow resistance—most commonly due to PH—leads to RV hypertrophy and dilation. Over time, however, this adaptive response becomes maladaptive, leading to progressive RV–arterial uncoupling and chronic RHF.

TR further increases RV volume overload, perpetuating a vicious cycle that accelerates RV deterioration and the progression of RHF [24,25]. As a consequence, RV filling increases along the passive portion of the diastolic pressure–volume relationship, and changes in initial myocardial fiber length (preload) modulate the myocardium’s ability to generate force. Although the RV acutely augments stroke volume through the Frank–Starling mechanism, its thin myocardium has limited capacity to counteract sustained elevations in load. Over time, chronic loading conditions disrupt this compensatory balance, leading to progressive RV–pulmonary arterial uncoupling, rising right-sided filling pressures, and finally prompting systemic venous congestion [26].

Patients who tolerate TR for longer periods are typically those with pulmonary pressures within normal limits. However, TR may also occur despite apparently normal PAP in advanced RHF, reflecting the exhaustion of RV compensatory capacity.

Chronic RHF mainly reflects the loss of the RV’s ability to accommodate venous return. This is frequently followed by a consequent deterioration in renal function [27]. Contrary to earlier beliefs, elevated right-sided filling pressures in chronic RHF do not represent a compensatory mechanism but rather signify the failure to reduce RA/CV pressures. Although the right-sided chambers and venous system can tolerate large fluid volumes, persistent visceral congestion leads to significant tissue injury.

Compared with the normal RV, elevated PAP imposes a substantial increase in energy demand to propel blood into the pulmonary circulation. When RV dilation and reduced stroke volume develop, energy requirements rise further, profoundly worsening RV metabolic balance and RV–arterial coupling. Ultimately, inadequate blood delivery to the LV can precipitate left-sided HF, a fall in CO, and potentially death [28].

A particularly illustrative example of these mechanisms is the development of RHF after LVAD implantation. In the LVAD setting, abrupt unloading of the LV exaggerates leftward septal shift, distorts RV geometry, and may worsen functional TR. At the same time, the increase in systemic output imposed by the device markedly raises venous return, which a vulnerable RV may be unable to accommodate. When RV forward flow becomes insufficient to sustain pulmonary perfusion, the LVAD itself becomes underfilled, resulting in low device flows and systemic hypoperfusion [29]. A similar susceptibility may be observed immediately after heart transplantation, when the donor RV is suddenly exposed to the recipient’s pulmonary vascular load. These scenarios highlight how mechanical interventions can precipitate acute right-sided decompensation and underscore the central role of RV–arterial coupling in maintaining circulatory stability.

## 4. Epidemiology and the Clinical Prognostic Burden

The diverse aetiologies and the absence of a standardized definition contribute to RHF being frequently underdiagnosed [30]. While its true prevalence is likely underestimated, RHF is highly prevalent across the HF spectrum, affecting both patients with reduced ejection fraction (HFrEF) and those with preserved ejection fraction (HFpEF). In the HFrEF population, the prevalence of RVD has been reported as high as 48%, while in HFpEF, RHF is increasingly recognized as a primary driver of clinical instability and long-term outcomes.

The prevalence of RVD and RHF increases with advancing left-sided heart disease, yet these conditions are not invariably irreversible. Indeed, RV function may improve following appropriate therapeutic interventions. Recovery of RV dysfunction has been documented in patients with dilated cardiomyopathy who underwent optimized medical therapy [31].

The syndrome may present as isolated RHF or as a combination of LV and RV failure, commonly referred to as biventricular failure. Post-capillary PH—whether occurring in isolation or in combination with a pre-capillary component—is a frequent complication of LHF [32,33,34].

**Figure 2:**
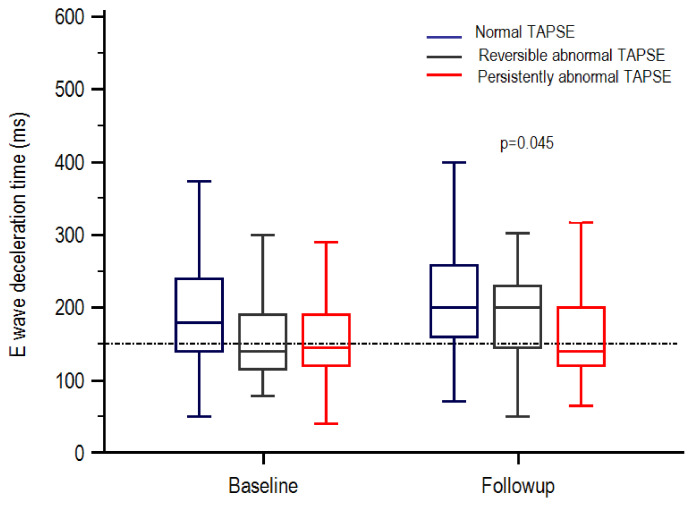
Long-term changes in mitral E-wave deceleration time (as a surrogate measure of LV filling pressure) from baseline to follow-up, stratified by right ventricular (RV) longitudinal function response, as assessed by tricuspid annular plane systolic excursion. Following left-sided medical unloading therapy, an improvement in RV systolic function was observed in patients exhibiting a prolongation of E-wave deceleration time (*p* = 0.045), underscoring the potential for LV filling pressure reduction and the reversibility of secondary RV dysfunction. Legend: TAPSE: tricuspid annular plane systolic excursion.

Data from the CHARITEM registry indicate that RHF, as the primary presentation of acute decompensated HF, accounts for 3–9% of hospital admissions [35]. However, RHF most commonly manifests as a secondary consequence of left-sided heart disease. Modern estimates suggest that left-sided filling pressures are a contributing factor in more than one-fifth of all decompensated RHF cases. This secondary progression is usually driven by the retrograde transmission of elevated left-sided filling pressures, leading to PH and a subsequent mismatch between RV contractility and pulmonary afterload—i.e., RV–arterial uncoupling. An improvement in RV function may be observed following the reduction in left-sided filling pressure by unloading therapies (Figure 2).

The prognosis for RHF remains highly variable, dictated by the underlying cause and the severity of clinical manifestations. While some historical data suggest that RHF carries a survival burden comparable to LHF, high-quality, dedicated prognostic data remain scarce. Traditionally, research has focused on echocardiographic markers of RVD rather than the clinical syndrome of RHF itself. However, contemporary evidence suggests that isolated systolic markers may be insufficient to predict outcomes. Signs and symptoms of congestion and systemic venous hypertension after 4 to 6 weeks of management have been associated with adverse prognosis in patients with advanced HF [36]. Therefore, the persistence of raised RA/CV pressures remains a critical hallmark of poor prognosis, emphasizing that the inability of the right heart to handle venous return is as lethal as the failure of the left heart to maintain CO [37,38].

## 5. From Clinical Manifestations to Diagnosis

Although current definitions of RHF tentatively describe the essential features of this syndrome, they still mirror the traditional left-sided framework and do not fully reflect its distinctive structural, haemodynamic and clinical characteristics. We believe that the peculiar characteristics of RHF—and the specific acute or chronic circumstances that give rise to the syndrome—should be more clearly delineated to fully convey the true nature of this disease state. Moreover, information derived from diagnostic tools, particularly echocardiography, which can substantially support the diagnostic process, should be integrated with clinical findings to achieve a more accurate characterisation of RHF [39].

Except for “de novo”cases, the syndrome is basically mediated by an elevation of RA and CV pressures [40,41]. From a mechanistic standpoint, chronic RHF is characterized by the inability of the heart to decongest the systemic venous system. The exhaustion of this adaptive mechanism leads to manifestations related to elevated venous pressure, including the rise in jugular vein pressure (JVP) [42]. As a result, fluid accumulates within the soft tissues. Swelling or pain in the upper abdomen is common, often resulting from fluid retention and congestion in the liver and gut. The classic signs typically follow, including:

1. Jugular venous distension (greater than 2 cm above the sternal angle);
2. Bilateral leg or ankle oedema;
3. Congestive hepatomegaly (and other congestive signs).

While pronounced peripheral oedema, anasarca, ascites, or liver distension are uncommon in acute settings, they frequently arise in decompensated chronic RHF. Signs and symptoms of RHF are listed in Table 4.

Although backward failure dominates the clinical picture in mild and moderate stages, forward flow impairment may coexist, particularly in advanced or biventricular disease. Severe RV dilation can compromise LV filling through ventricular interdependence, leading to left-sided underfilling, reduced CO, and systemic hypoperfusion. Importantly, while forward failure contributes to clinical severity, it is not specific to RHF and therefore cannot serve as a diagnostic criterion.

Given the heterogeneity of presentations, the diagnosis of chronic RHF requires the integration of clinical assessment with objective evidence of RV overload and/or dysfunction. Echocardiography plays a central role, providing non-invasive evaluation of RA/CV pressures, RV systolic performance, pulmonary pressures, and RV–pulmonary arterial coupling. When combined with clinical findings, these parameters allow a more accurate and reproducible characterization of the syndrome.

## 6. The Role of Biomarkers

Circulating concentrations of NP—BNP or its N-terminal fragment (NT-proBNP)—rise in direct response to increased right-sided filling pressures and myocardial wall stress [43,44]. In the context of RV overload—often driven by PH or valvular disease—these peptides act as critical physiological antagonists to maladaptive neurohormonal activation. In patients with RHF, their increased levels reflect the severity of both haemodynamic impairment and congestion and serve to identify individuals at increased risk of clinical deterioration or RHF progression, even when peripheral oedema or symptoms appear to be negligible. The presence of RVD in patients with pre-existing LV systolic dysfunction (ejection fraction < 40%) was associated with higher plasma BNP [45]. In patients with HFrEF, the combination of compromised RV function, as assessed by tricuspid annular plane systolic excursion (TAPSE), with echocardiographic evidence of increased RA pressure identified those with the higher NTproBNP levels and the worst prognosis [46].

## 7. Diagnostic Criteria

The diagnosis of right-sided heart failure is more challenging than that of LHF and requires a high degree of clinical suspicion, as the syndrome is frequently underrecognized or identified late, with adverse consequences for the patient’s quality of life and prognosis. Establishing the diagnosis relies on the careful integration of medical history and physical examination with objective evidence of RV overload and/or dysfunction and systemic congestion.

Expert consensus recommendations from the 2024 HFA and the EAPCI of the ESC clinical consensus statement advocate a comprehensive diagnostic strategy that incorporates clinical findings, echocardiographic assessment, laboratory markers, and haemodynamic measurements [7]. With the exception of acute “de novo” RHF, presence of elevated RA/CV pressures is generally considered an essential diagnostic criterion with important prognostic implications [47]. Information on right-sided filling pressures can be obtained clinically—through assessment of JVP (neck vein distension, positive hepatojugular reflux)—or echocardiographically, by identifying a dilated inferior vena cava (IVC) with reduced or absent respiratory variation. Filling pressures are considered normal or low when the IVC diameter decreases by ≥50% with inspiration, whereas an inspiratory collapse <50% indicates elevated RA/CV pressures [48]. Echocardiographic assessment of right-sided filling pressures predicted the outcome of patients with PH [49].

Echocardiography is the most widely available imaging modality for evaluating RV systolic function. The diagnosis of RV dysfunction relies on quantitative assessment of global RV performance using at least one of the following parameters: TAPSE, fractional area change (FAC), Doppler tissue imaging–derived systolic (S′) velocity of the tricuspid annulus, or RV free-wall longitudinal strain (RVFWLS) by 2D speckle-tracking echocardiography [50,51]. TAPSE is the most commonly used parameter, with a lower reference limit of <17 mm.

Echocardiography is also central to the assessment of RV overload and PH in clinical practice. TR velocity (TRV), which reflects the systolic pressure gradient between the RV and RA, is typically measured using continuous-wave Doppler. A TRV > 2.8 m/s is generally considered indicative of PH. Pulmonary artery systolic pressure (PASP) can be estimated by adding echocardiographically-derived RA pressure to the transtricuspid gradient derived from TRV. Combined indices such as the TAPSE/PASP ratio provide valuable information on RV–pulmonary arterial coupling [52].

The centrality of elevated right-sided filling pressures in defining RHF is supported by the INTERMACS consensus [53], which identifies RA/CV pressures > 16 mmHg as the mandatory first criterion for diagnosing RHF, whether measured invasively or inferred from jugular venous distension or IVC dilation. This heamodynamic abnormality is not merely a marker of elevated venous pressure and congestion but also a pathophysiological driver of organ dysfunction. In a landmark analysis of patients with cardiovascular disease, elevated CV pressure has been shown to correlate strongly with impaired renal function and increased mortality, underscoring that systemic venous hypertension represents a maladaptive and prognostically adverse state rather than a compensatory response [54]. Likewise, disproportionate right-sided filling pressures—particularly when RA pressure exceeds left-sided filling pressure—identify a distinct phenotype of true right-sided failure, characterised by renal dysfunction and a markedly increased risk of death [55]. Together, these findings reinforce that presence of elevated RA/CV pressures are the fundamental haemodynamic signature of RHF, providing a robust physiological and prognostic rationale for its role as an essential major criterion in the diagnostic algorithm.

## 8. A New Diagnostic Algorithm

Based on these considerations, clinical–echocardiographic criteria for diagnosing chronic RHF can be proposed. Here, the clinical challenge is not immediate forward failure and circulatory collapse, but chronic backward failure. While existing scoring systems such as the Right Ventricular Failure Risk Score (RVFRS) [56] and the EUROMACS-RHF score [57] provide useful prognostic information regarding the risk of developing RHF, they were not designed as diagnostic tools for establishing the presence of RHF. Rather, they were developed to identify patients at increased risk of postoperative or future RHF, particularly in advanced HF populations undergoing mechanical circulatory support. In contrast, a structured diagnostic framework suitable for identifying and stratifying chronic RHF is still lacking. To address this gap, we have designed a dedicated pragmatic diagnostic algorithm.

**Figure 3:**
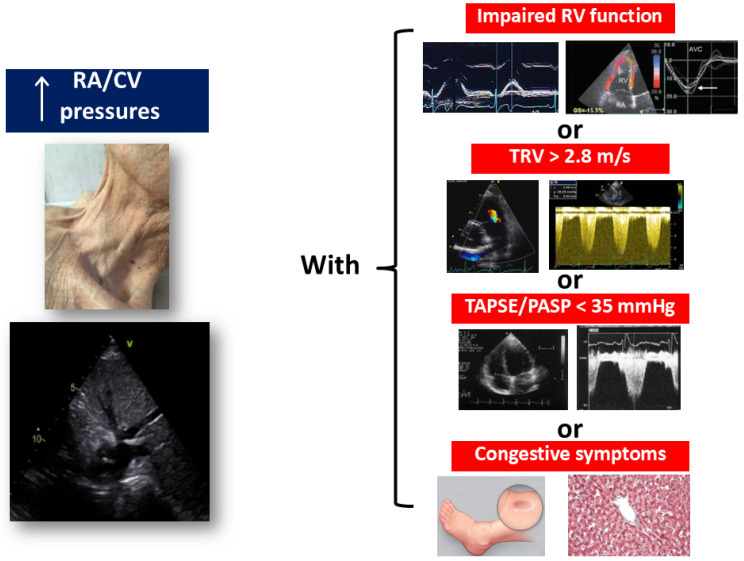
Diagram of the right heart failure (RHF) diagnostic algorithm. The blue text box (**left**) denotes the mandatory major criterion of elevated right atrial and central venous pressures (indicated by the upward white arrow), clinically illustrated by jugular venous distension (**top**) and a dilated inferior vena cava (**bottom**). Linked by a black grouping brace, the red text boxes (**right**) display the minor criteria, where the presence of at least one parameter is required (“or”). Internal graphic markers include: multi-colored lines in the speckle-tracking panel representing distinct right ventricular myocardial segments; an internal white arrow pointing to the peak systolic strain reduction; and continuous-wave/spectral Doppler tracings used to quantify tricuspid regurgitation velocity (TRV), tricuspid annular plane systolic excursion (TAPSE), and pulmonary artery systolic pressure (PASP).

**Figure 4:**
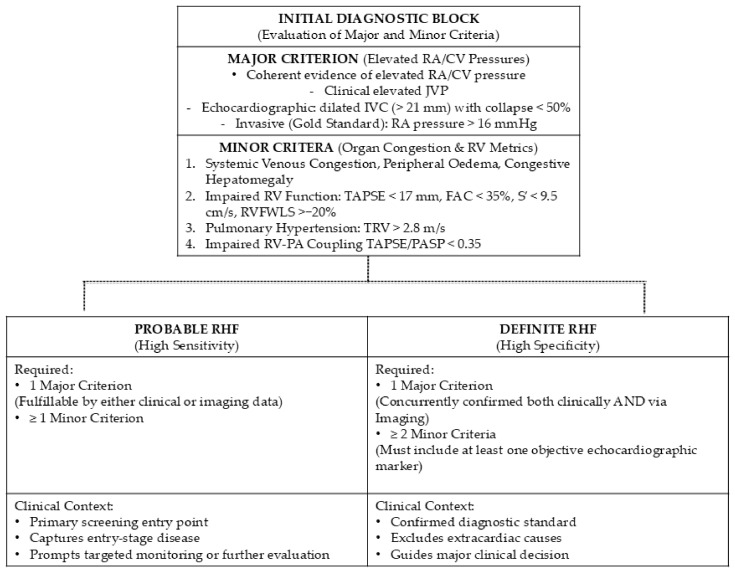
Hierarchical multiparametric diagnostic framework for chronic right heart failure (for description see the text). Legend: CV: central venous; FAC: fractional area change; IVC: inferior vena cava; JVP: jugular venous pressure; PASP: pulmonary artery systolic pressure; RA: right atrial; RHF: right heart failure; RV: right ventricular; RVFWLS: left ventricular free wall longitudinal strain; RV-PA: right ventricular–pulmonary arterial; S′: tissue Doppler-derived tricuspid annular systolic velocity; TAPSE: tricuspid annular plane systolic excursion; TRV: tricuspid regurgitation velocity.

The algorithm includes one major criterion and minor criteria (Figure 3 and Figure 4):

The major criterion includes:

- Evidence of increased RA/CV pressures, assessed clinically by elevated JVP and echocardiographically using IVC diameter and respiratory variation as surrogates of RA pressure.

The major criterion requires coherent and convergent evidence of elevated RA/CV pressures. Because individual clinical signs—such as jugular venous distension or a positive hepatojugular reflux—may have limited accuracy and are susceptible to interobserver variability, they cannot be considered sufficient in isolation, particularly when echocardiographic surrogates do not support the occurrence of elevated right-sided filling pressures. For example, an apparently distended jugular vein in the presence of a small, normally collapsing IVC (e.g., diameter < 10 mm) should not be interpreted as fulfilling the major criterion. Instead, the diagnosis requires either unequivocal echocardiographic evidence (dilated IVC with reduced inspiratory collapse) or clinical evidence of raised JVP.

When available, invasive confirmation of elevated right-sided filling pressures using the INTERMACS threshold of RA pressure > 16 mmHg should be considered the haemodynamic gold standard for fulfilling the major criterion [53].

Minor criteria comprise:

- peripheral oedema and congestive symptoms (such as congestive epatomegaly);
- impaired RV function, such as TAPSE < 17 mm, FAC < 35%, S′ < 9.5 m/s; RVFWLS > −20%.
- pulmonary hypertension, reflected by TRV > 2.8 m/s.
- Impaired RV-arterial coupling: TAPSE/PASP < 0.35 mm/mmHg.

The minor criteria highlight that elevated RA/CV pressures do not always signify true RHF—for example, in patients with expanded blood volume from renal sodium and water retention. The dual-tier structure helps avoid misclassification in complex phenotypes such as liver cirrhosis or primary venous insufficiency, where lower-limb oedema or ascites commonly occur despite entirely normal right-sided pressures. Conversely, it ensures that subtle backward failure—often obscured by aggressive diuretic therapy—is still recognized through imaging markers such as a partially uncollapsed IVC combined with reduced TAPSE, FAC or RVFWLS and/or TRV > 2.8 m/s.

## 9. Limitations of the Diagnostic Algorithm

A major challenge in the clinical appraisal of chronic RHF is its symptomatic overlap with several non-cardiac and extracardiac conditions. For example, bilateral peripheral oedema and systemic fluid retention are shared features of severe chronic venous insufficiency, liver cirrhosis, and advanced renal failure.

Clinical and echocardiographic surrogates of RA/PV pressures—such as jugular venous distension, hepatojugular reflux, and IVC dilation—are essential components of bedside assessment, yet each carries inherent limitations. Their accuracy may be reduced in obesity, lung hyperinflation, mechanical ventilation, or in patients with abnormal venous compliance. For this reason, the diagnostic algorithm emphasises integration rather than reliance on any single sign, and discordant findings should prompt careful reassessment or haemodynamic confirmation when clinically appropriate.

While forward-flow impairment (e.g., low CO, systemic hypotension, or peripheral hypoperfusion) represents a key pathophysiological consequence of advanced RHF and biventricular dysfunction, its clinical utility as a diagnostic criterion for chronic RHF is intrinsically limited. In the chronic setting, signs of forward failure lack sufficient specificity, as they are frequently confounded by, or indistinguishable from, primary LV failure or concomitant biventricular exhaustion. Consequently, to preserve the diagnostic specificity of the proposed algorithm and avoid overdiagnosis, forward failure has not been included as a standalone minor criterion. Instead, it should be regarded as a crucial ‘red flag’ signaling advanced disease progression and impending RV-arterial uncoupling in patients with already established right-sided congestion.

## 10. Definite vs. Probable RHF

To balance diagnostic sensitivity with specificity, the proposed framework stratifies patients into two hierarchical categories: Probable RHF and Definite RHF (Figure 4). A two-tier diagnostic approach is necessary because chronic RHF exists along a spectrum of diagnostic certainty, and no single clinical, echocardiographic, or haemodynamic finding is sufficiently sensitive and specific across all practice settings. Separating Probable from Definite disease allows early identification of patients who may benefit from further evaluation while maintaining a higher evidentiary threshold for establishing a confirmed diagnosis when more complete and objective data are available. This distinction reflects not only the need to differentiate screening from confirmatory diagnoses, but also a common practical limitation in the evaluation of chronic RHF: complete assessment of both clinical and echocardiographic markers of elevated right-sided filling pressures is frequently unavailable or unreliable.

In routine practice, jugular venous examination may be obscured by obesity, neck anatomy, oedema, or poor patient positioning, whereas echocardiographic visualization of the IVC and RV may be limited by chronic obstructive pulmonary disease, poor acoustic windows, mechanical ventilation, or suboptimal image quality. Consequently, many patients cannot be confidently assessed using all components of the diagnostic algorithm at a single time point. The two-tier framework acknowledges this reality by providing a sensitive screening category when only partial information is available, while reserving a more stringent category for cases in which comprehensive objective confirmation can be obtained.

The first category, Probable RHF (high sensitivity), functions primarily as a screening diagnosis. It is intended for patients in whom only clinical findings, only echocardiographic findings, or an incomplete combination of both are available for assessment. In this setting, the presence of one major criterion (either clinical or echocardiographic evidence of elevated RA/CV pressures) plus one minor criterion identifies patients with a high likelihood of right-heart decompensation who warrant further evaluation, closer monitoring, or targeted treatment. This simplified “1 + 1” approach prioritizes sensitivity and minimizes the risk of overlooking clinically significant RHF during initial assessment.

However, because this threshold can be fulfilled using incomplete datasets and by combinations of findings that are not specific to RV failure, Probable RHF should not be regarded as definitive proof of the occurrence of the syndrome. For example, a patient with elevated JVP and peripheral oedema may satisfy the screening criteria despite having congestion primarily driven by renal disease, isolated left-sided HF, severe TR with preserved RV contractility, or other non-cardiac causes of volume overload. Therefore, the Probable RHF category serves as an entry point for diagnostic consideration rather than a final diagnosis.

The second category, Definite RHF (high specificity), is designed for situations in which adequate clinical and imaging data are available to establish a robust diagnosis. This category requires concordant evidence of elevated RA/CV pressures from both clinical and echocardiographic assessment (or invasive haemodynamic confirmation), together with at least two minor criteria, including mandatory echocardiographic evidence of RV dysfunction or overload. By requiring confirmation across complementary modalities and objective proof of RV impairment, this tier substantially reduces the risk of misclassifying extracardiac volume overload or isolated venous congestion as right HF. Consequently, Definite RHF represents the preferred diagnostic standard for clinical decision-making, research enrollment, and epidemiological studies.

This hierarchical approach allows clinicians to maintain high screening sensitivity when complete data are unavailable while preserving high diagnostic specificity when comprehensive evaluation can be performed. Patients meeting criteria for Probable RHF can undergo further imaging, haemodynamic evaluation, or longitudinal reassessment, whereas those fulfilling Definite RHF criteria have objective evidence linking elevated venous pressures and systemic congestion directly to the ongoing syndrome.

## 11. Potential Role of Biomarkers Within the Diagnostic Algorithm

Although biomarkers are not included among the core diagnostic criteria for chronic RHF, they may provide important complementary information. Elevated NP (BNP or NT-proBNP) reflect increased myocardial wall stress and right-sided filling pressures and correlate with the severity of RV dysfunction and haemodynamic congestion. Consequently, elevated peptide levels may support the diagnosis in patients with equivocal clinical/echo findings or in those receiving intensive diuretic therapy, where peripheral congestion is partially masked. Biomarkers may be particularly useful in situations where the major criterion of elevated RA/CV pressures is present, but the significance of accompanying minor criteria remains uncertain. For example, elevated NT-proBNP concentrations in a patient with a dilated, poorly collapsible IVC and mildly reduced TAPSE would strengthen the likelihood that these findings reflect true RHF rather than isolated volume overload. Conversely, normal NP levels would make clinically significant RHF less likely and should prompt consideration of alternative causes of oedema or venous congestion.

Beyond diagnosis, biomarkers provide valuable prognostic information. Elevated BNP, NT-proBNP, high-sensitivity cardiac troponins, soluble ST2, and galectin-3 have all been associated with worse RV function, progressive RV remodelling, increased hospitalization rates, and mortality. Their incorporation into the diagnostic pathway could therefore facilitate early risk stratification and identify patients who may benefit from closer monitoring or more aggressive treatment [14].

Nevertheless, biomarker concentrations may be influenced by age, renal dysfunction, obesity, atrial fibrillation, and concomitant left-sided heart disease. Therefore, biomarkers should not be considered standalone diagnostic markers for chronic RHF but rather adjunctive tools that complement clinical assessment and echocardiographic evaluation.

## 12. Conclusions and Future Directions

Chronic RHF remains an underecognized and inconsistently defined syndrome, largely because its clinical and haemodynamic foundations have historically been interpreted through a left-sided framework. The diagnostic algorithm proposed in this review aims to shift that paradigm by anchoring the definition of chronic RHF to its true haemodynamic core: persistent venous hypertension driven by elevated RA/CV pressures, rather than isolated abnormalities of RV systolic performance. By integrating this central concept with objective markers of systemic congestion, RV dysfunction, PH, and RV–pulmonary arterial uncoupling, the algorithm provides a coherent, pragmatic structure that can be readily applied in routine clinical practice.

Future research should focus on prospectively validating this criteria-based approach across diverse aetiologies and care settings, assessing its prognostic utility, and determining whether earlier recognition of right-sided myocardial failure can improve therapeutic decision-making and patient outcomes. Ultimately, operationalizing RHF as a syndrome defined by backward failure and systemic venous hypertension may help harmonize clinical practice, refine patient phenotyping, and support the development of targeted interventions for this vulnerable population.

## Figures and Tables

**Table 1:** Right heart failure can be categorized into pulmonary vascular disease-related RHF, left heart disease-related RHF and primary right ventricular disorders.

Feature	Pulmonary Vascular Disease-Related RHF	Left Heart Disease-Related RHF	Primary Right Ventricular Disorders
Primary pathophysiology	Severe chronic pressure overload due to pulmonary vascular remodelling or obstruction (PAH and cor pulmonale)	Backward transmission of elevated left-sided filling pressure	Direct intrinsic myocardial injury
Echocardiographic & haemodynamic findings	PASP, PAMP marked elevated PCWP normal	PASP, PAMP moderately to severely elevated PCWP elevated	PASP, PAMP normal or moderately elevated PCWP normal
ECG	RV hypertrophy/strain patterns	Detects LV disease, AF, prior MI	Disease-specific patterns
MRI/CMR	Shows RV pressure overload remodelling	Demonstrated primary left-sided abnormalities	Best test for intrinsic RV myocardial disease
CT	Excellent for pulmonary vasculature and lung disease	Shows left heart enlargement and coronary disease	Can identify structural RV abnormalities less specific than MRI

Legend: CMR: cardiovascular magnetic resonance; CT: computed tomography; PAMP: mean pulmonary pressure; PASP: pulmonary artery systolic pressure; MRI: magnetic resonance imaging; PCWP: pulmonary capillary wedge pressure; PAH: pulmonary arterial hypertension; RHF: right heart failure; RV: right ventricular.

**Table 2:** Mechanisms and aetiolgies of right ventricular failure.

Mechanism	Aetiologies
Increased afterload	PH associated with HFrEF, HFmrEF and HFpEF
	Mitral stenosis
	Heart transplant and LV assist device
	Acute pulmonary embolism and chronic pulmonary thromboembolism
	Acute respiratory distress syndrome
	COVID-19
	PAH
	Chronic pulmonary disease
	Sleep-related breathing disorders Eisenmenger syndrome
Abnormal preload	Hypo- or hypervolemia
	Pericardial tamponade
	Mechanical ventilation
	Left-to-right shunt Lead-induced tricuspid regurgitation Congenital heart diseases with pulmonary overflow
Reduced contractility	RV ischemia/infarction
	Cardiomyopathies
	Myocarditis
	Arrhythmogenic RV cardiomyopathy Oncologic cardiotoxicity

Legend: HFrEF: HF with reduced EF, HFmrEF: HF with mildly reduced EF, HFpEF: HF with preserved EF; LV: left ventricular; PAH: pulmonary arterial hypertension; PH: pulmonary hypertension; RV: right ventricular.

**Table 3:** Comparative features of acute and chronic right heart failure.

Feature	Acute RHF	Chronic RHF
Common Etiologies	Massive PE, RV myocardial infarction, acute myocarditis	Pulmonary hypertension, LHF (HFrEF/HFpEF), Chronic valvular disease
Primary Mechanism	Sudden afterload mismatch or loss of contractility	Progressive RV remodelling and chronic pressure/volume overload
Dominant Clinical Sign	Hypotension/Cardiogenic shock (low cardiac output)	Systemic congestion (oedema, ascites, hepatomegaly)
RV Morphology	Normal size or acutely dilated; thin-walled	RV hypertrophy (thickened wall) and marked dilation
RA Pressure	Rapid rise (often leads to immediate syncope/collapse)	Chronic elevation (leads to organ congestive dysfunction)
Adaptation	No time for compensation; high risk of death	RV remodelling (initially adaptive, eventually maladaptive)

Legend: HfrEF: HF with reduced EF; HFpEF: HF with preserved EF; LHF: left-sided HF; PE: pulmonary embolism; RHF: right heart failure; RA: right atrial; RV: right ventricular.

**Table 4:** Symptoms and signs of right-sided heart failure.

More Frequent	More Specific
Shortness of breath and fatigue	Hepatojugular reflux
Palpitations	Kussmaul sign
Systemic venous hypertension	Holosystolic murmur with Rivero-Carvalho sign
Neck vein distension with jugular turgor	Right ventricular gallop with third sound
Peripheral oedema	Ascites
Congestive hepatomegaly	Hydrothorax
Swelling and/or pain of the upper abdomen	Anorexia, nausea, and abdominal pain
	Malnutrition and cachexia

## Data Availability

No new data were created or analyzed in this study. Data sharing is not applicable to this article.
